# Acceptance of vaccination against pertussis, COVID-19 and influenza during pregnancy: a cross-sectional study

**DOI:** 10.1186/s12884-023-05505-9

**Published:** 2023-03-30

**Authors:** Veja Widdershoven, Rianne P. Reijs, Annika Eskes, Amanja Verhaegh-Haasnoot, Christian J.P.A. Hoebe

**Affiliations:** 1 Department of Social Medicine, Care and Public Health Research Institute (CAPHRI), Maastricht University, PO Box 616, 6200 MD Maastricht, The Netherlands; 2 Department of Sexual Health, Infectious Diseases and Environmental Health, Living Lab Public Health South, South Limburg Public Health Service, PO Box 33, 6400 AA Heerlen, The Netherlands; 3 Department of Youth Health Care, Living Lab Public Health South, Public Health Service South Limburg, Heerlen, The Netherlands; 4 Department of Youth Health Care, Public Health Service Hollands Noorden, Alkmaar, The Netherlands; 5 Department of Medical Microbiology, Care and Public Health Research Institute (CAPHRI), Maastricht University Medical Centre (MUMC+), PO Box 5800, 6202 AZ Maastricht, The Netherlands

**Keywords:** Vaccination refusal, Vaccination Coverage, Pregnancy, Whooping cough, COVID-19 vaccines, Influenza vaccines

## Abstract

**Background:**

This study aims to assess the uptake of maternal pertussis and COVID-19 vaccination and the intention towards accepting the maternal influenza vaccination. Insights into different socio-demographic factors related to maternal vaccination coverage might help to address vaccine acceptance and improve maternal vaccine uptake in the future.

**Methods:**

We conducted a cross-sectional survey among pregnant women and recent mothers, up to 6 months post-partum. The primary outcome measures of this study were behaviour for maternal pertussis and COVID-19 vaccination, and maternal influenza vaccination intention. Associations between socio-demographic factors and maternal pertussis vaccination and maternal COVID-19 vaccination behaviour; and socio-demographic factors and maternal influenza vaccination intention were assessed using binary logistic regression analyses.

**Results:**

In total 1361 respondents filled out the questionnaire. Almost all women (95%) were vaccinated against pertussis during pregnancy, while almost two-third were vaccinated against COVID-19 during pregnancy (58%) and almost one-third (28%) had a positive intention towards receiving the maternal influenza vaccination. Results show that young maternal age and low education level were associated with lower maternal vaccination acceptance.

**Conclusion:**

Vaccination campaigns focusing on the severity of diseases that are prevented, are needed to increase maternal vaccine acceptance in younger and low-educated pregnant women. We expect that differences in vaccination coverage between the three maternal vaccinations might partly be explained by existing recommendations, campaigns and whether the vaccination is part of the national immunisation program.

**Supplementary Information:**

The online version contains supplementary material available at 10.1186/s12884-023-05505-9.

## Background

Maternal immunisation is a relatively new strategy that can provide protection against sequelae from a number of infectious diseases for mainly the newborn, mainly the mother or both [1–3]. The maternal pertussis vaccination is an example of maternal immunisation that helps to protect the newborn from severe pertussis in their first months of life [4, 5], maternal COVID-19 vaccination mainly helps to protect the pregnant mother against severe COVID-19 outcomes and maternal influenza vaccination is an example which helps to protect both pregnant women and their newborn against severe influenza outcomes. Because of the benefits of maternal immunisation, the World Health Organization (WHO) recommends pertussis, COVID-19 and influenza vaccination for pregnant women [1, 2, 6].

In December 2019, the maternal pertussis vaccination was introduced in the Dutch National Immunisation Program while introduction was earlier in the UK (2012), Australia (2015), Belgium (2013) and the US (2012) [6–10]. In 2021, maternal pertussis vaccination coverage in the Netherlands was about 66% [11]. Studies from other countries showed similar uptake; about 68% in the UK in 2020, 54% in the US in 2020 and 85% in Australia in 2017 [6, 12–14]. A number of demographic factors that are related to maternal pertussis vaccination refusal have been reported in previous studies, such as low socioeconomic status (SES), low education level, migration status and religious background [15–18].

Since the global COVID-19 outbreak, pregnant women are also advised to get vaccinated against COVID-19 because they have a higher chance of severe illness with COVID-19 disease [19, 20]. In the US, the American College of Obstetricians and Gynaecologists (ACOG) already advised to vaccinate all pregnant women in January 2021. In England and the Netherlands, this advice was implemented in April 2021 [21]. Previous studies indicated lower COVID-19 vaccination coverages among pregnant women compared to the general female population of reproductive age [19, 20]. Two studies in the UK reported that between 29% and 43% of pregnant women received at least one dose of COVID-19 vaccination [22, 23]. Studies in the UK and Scotland showed that pregnant women of younger age (below 25 years old) were less likely to accept the COVID-19 vaccination [19, 22, 23].

Whereas implementation of the maternal pertussis vaccination already took place in most European countries, this is not the case for the maternal influenza vaccination. In the Netherlands, the maternal influenza vaccination has been recommended by the Dutch health council since 2021, but the vaccination isn’t introduced in the national immunisation program (NIP) yet and there are no campaigns to provide information about this vaccination. Therefore, pregnant women might not be aware of the vaccination and the recommendation. These women can take initiative to make an appointment for the maternal influenza vaccination at their general practitioner. However, previous studies reported that access issues might be an important barrier to maternal influenza vaccination [24]. Therefore, only very small numbers of pregnant women in the Netherlands are vaccinated. In the US and England, the uptake of maternal influenza vaccination between 2019 and 2020 was respectively 61% and 44% [2]. A previous study from Barrett et al. [25] determined that higher socioeconomic status and education level were associated with higher maternal influenza vaccination acceptance. Furthermore, receiving information from a health professional and wanting to protect the baby were main reasons for pregnant women to accept the maternal influenza vaccination [25]. Common reasons for refusing the maternal influenza vaccination were: low perceived susceptibility, concern about harming the baby and themselves [25].

Insights into different socio-demographic factors related to maternal pertussis, COVID-19 and influenza vaccination coverage might help to address vaccine acceptance and improve maternal vaccine uptake in the future. This study aims to assess behaviour or intention of three maternal vaccinations (1) pertussis vaccination behaviour, (2) COVID-19 vaccination behaviour and (3) influenza vaccination intention. Because intention was used as a measure of maternal influenza vaccination behaviour, we describe main reasons for predicted maternal influenza vaccination acceptance or refusal. Secondly, we examined which socio-demographic factors are related to the acceptance of maternal vaccination for the three different vaccines.

## Methods

### Study design and setting

We performed a large cross-sectional online questionnaire study among pregnant women and recent mothers, up to 6 months post-partum. Questionnaires were distributed at three Youth Health Care services in the Netherlands between September 2021 and May 2022. All parents of newborns in the Netherlands are actively invited to visit the Youth Health Care clinic. Virtually all parents accept this invitation in the first year of their child’s life. Both pregnant women and recent mothers received an invitation leaflet containing information about the study and the invitation for the study questionnaire. Two Youth Health Care services sent reminders via e-mail after the invitation, respectively 2 and 4 weeks after the consultation. Besides the invitation leaflet, assisted questionnaires were used at one Youth Health Care location to increase response rates. In addition, the questionnaire was nationally distributed via social media channels, including Facebook, Instagram and Twitter.

### Participants

All women 18 years and older, being pregnant or up to 6 months postpartum and being able to answer the questions in Dutch were eligible for participation. At the location where assisted questionnaires were used, women who were able to answer the questions in English could also be included. Women under 18 years of age and women who did not give consent to fill in the questionnaire were excluded. About 3500 women received an invitation to fill out the questionnaire, excluding the distribution via social media, based on the number of reminders that were sent. Completing the questionnaire was voluntary and based on written informed consent.

### Data collection

The primary outcome measures of this study were behaviour for maternal pertussis vaccination, behaviour for maternal COVID-19 vaccination and maternal influenza vaccination intention. Maternal pertussis and COVID-19 vaccination behaviour were measured with one question each: ‘*Did you accept the maternal pertussis /COVID-19 vaccination during pregnancy?’ (1 = No, 2 = Yes).* Intention towards maternal influenza vaccination was measured with the question: ‘*If the influenza vaccination was offered to pregnant women right now, would you accept it?*’ (*1 = No, never* to *5 = Yes, always)*. Socio-demographics included age category, highest completed education (low, medium or high) and country of birth (Dutch or other). The classification of education levels was based on the International Standard Classification of Education [26]. Furthermore, general vaccination beliefs was included as an attitudinal factor and measured with three items, for example ‘*Vaccinations are important for staying healthy’ (*5-point Likert scales with end-points labelled as *1 = totally disagree* and *5 = totally agree)*. Because of sufficient internal consistency (Cronbach’s Alpha α = 0.694) these three items were combined. Additionally, the questionnaire included two multiple choice questions about the reasons behind accepting or refusing the maternal influenza vaccination in the future.

### Influence of COVID-19 vaccination advice for pregnant women

In the Netherlands, the advice to vaccinate all pregnant women against COVID-19 was implemented in April 2021. Before April 2021, the COVID-19 vaccination for pregnant women was not encouraged. Therefore, we checked whether this change in advice affected the acceptance of COVID-19 vaccinations during pregnancy among women who filled in the questionnaire between September 2021 and January 2022 (nine months after April 2021). The data showed no significant differences between women who filled in the questionnaire between September 2021 and January 2022 (early stage of the study) and women who filled in the questionnaire after January 2022 (last stage). Therefore no participants were excluded from further analyses as no bias was expected during different times of the study period.

### Statistical analysis

Prior to the main analyses, we conducted a sensitivity analysis to explore a difference in COVID-19 vaccination behaviour between women who filled in the questionnaire between September 2021 and January 2022 (early stage of the study) and women who filled in the questionnaire after January 2022 (last stage). Because no significant difference was found, all participants were included in the main analyses.

Descriptive analyses (frequencies) on demographic variables and determinants were performed to provide an overview of the sample. Maternal influenza vaccination intention was classified in two groups: low intention (0 = 1.0–3.0) and high intention (1 = 4.0–5.0). Associations between socio-demographic factors and maternal pertussis vaccination and maternal COVID-19 vaccination behaviour; and socio-demographic factors and maternal influenza vaccination intention were then assessed using binary logistic regression analyses. Multicollinearity between factors was checked (VIF < 5). Questions about main reasons for accepting or refusing maternal influenza vaccination in the future were analysed using descriptive analyses. A p-value < 0.05 was considered statistically significant. All analyses were performed using IBM SPSS Statistics version 26.0 (IBM; Armonk, New York, USA, 2022).

### Ethical review

The study protocol, participant information form and written informed consent form were approved by the Medical Ethical Committee of Maastricht University Medical Centre in Maastricht, the Netherlands (METC 2020–2296). Obtained data were not traceable to individuals and were analysed anonymously.

## Results

In total 1361 respondents filled out the questionnaire The estimated response rate was 39%, based on the number of invitations sent by the Youth Health Care services. The majority of the respondents (1266; 93%) were recruited through the Youth Health Care services, 54 through social media and 41 through assisted questionnaires. As shown in Table 1, the majority of the respondents were of Dutch origin (95%) and had a high education level (67%). Most participants (81%) were between 25 and 35 years old. The age distribution of our study population is comparable with all Dutch pregnant women with a mean age of 30.3 years for their first child and 32.3 years for their second child in 2021 [27]. Except for the lowest age category (18–24 years), most women were highly educated (between 60 and 76%), followed by moderate and low education level. Within the lowest age category (18–24 years), 26.0% had a low education level, 58.0% had a moderate education level and only 16.0% was highly educated.

**Table 1:** Characteristics of the respondents

	All participants (*N* = 1361)	
Demographic factors	n/N	%
Age		
*18–24 years* *25–30 years* *31–35 years* *36–40 years* *40 + years*	50/1361 532/1361 576/1361 178/1361 25/1361	3.7 39.1 42.3 13.1 1.8
Education		
*Low* *Intermediate* *High*	57/1361 395/1361 909/1361	4.2 29.0 66.8
Country of birth		
*Dutch* *Other*	1286/1361 75/1361	94.5 5.5
**Personal factors**		
Pertussis vaccination		
*Yes* *No*	1294/1361 67/1361	95.1 4.9

### Pertussis vaccination behaviour

The majority of women (95%) were vaccinated against pertussis during pregnancy. Univariate logistic regression showed a significantly higher odds of accepting the maternal pertussis vaccination among participants with an intermediate (OR = 3.79, 95%CI [1.79, 8.202]) or high (OR = 8.09, 95%CI [3.88, 16.90]) education level compared to participants with a low education level (Table 2). Furthermore, being more positive about vaccinations in general was related to higher maternal pertussis vaccination acceptance (OR = 4.60, 95%CI [3.20, 6.60]). In contrast, participants born outside the Netherlands (OR = 0.27, 95%CI [0.13, 0.53]) and younger participants were less likely to accept the maternal pertussis vaccination (OR = 0.26, 95%CI [0.10, 0.63]).

### COVID-19 vaccination behaviour

More than half of the women (58%) were vaccinated against COVID-19 during their pregnancy. Univariate logistic regression showed that high education level compared to participants with a low education level was positively associated with COVID-19 vaccination acceptance (OR = 2.18, 95%CI [1.27, 3.74]) (Table 2). Moreover, two age categories were negatively associated with COVID-19 vaccination acceptance: younger participants (18–24 years old (OR = 0.39, 95%CI [0.21, 0.70]) and 25–30 years old (OR = 0.60, 95%CI [0.47, 0.76])) had a lower odds of COVID-19 vaccination acceptance compared to participants between the age of 31 and 35 years old. Women who refused maternal pertussis vaccination were also less likely to accept the COVID-19 vaccination (OR = 0.18, 95%CI [0.10, 0.32]). Furthermore, being more positive about vaccinations in general was related to higher COVID-19 vaccination acceptance (OR = 3.14, 95%CI [2.60, 3.79]).

### Influenza vaccination intention

**Fig. 1:**
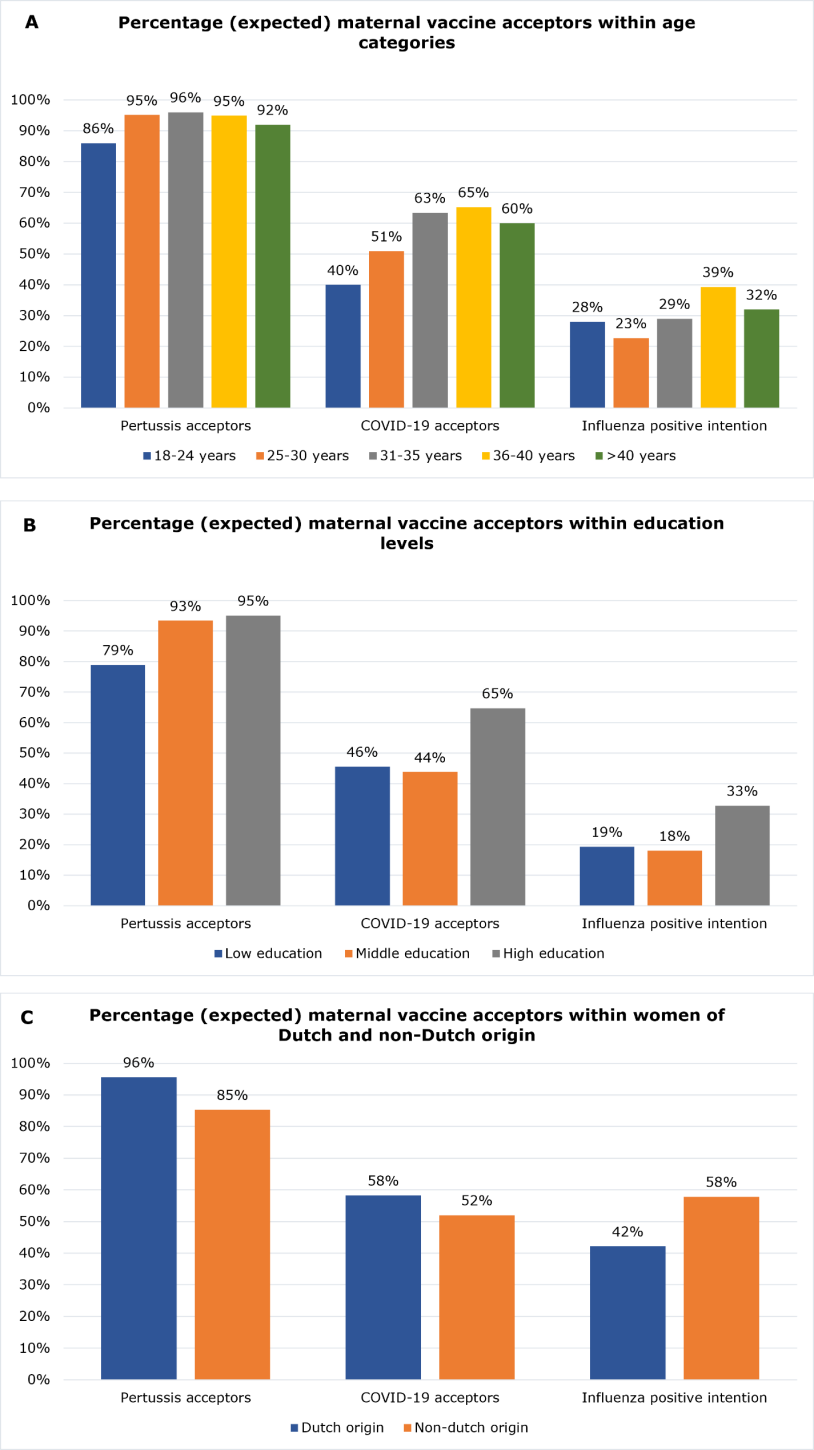
Percentages of women that accepted maternal pertussis and COVID-19 vaccination and had a positive intention towards maternal influenza vaccination among different categories

Less than one-third (28%) of the women indicated having a high intention towards receiving the maternal influenza vaccination if it would be recommended and offered to all pregnant women. Univariate logistic regression showed a significantly higher odds of having a high intention for maternal influenza vaccination among participants with a high education level (OR = 2.04, 95%CI [1.04, 4.00]) and participants between the age of 36 and 40 years old (OR = 1.59, 95%CI [1.12, 2.25]). On the other hand, just like the maternal pertussis vaccination and COVID-19 vaccination, younger participants had a lower odds compared to participants between the age of 31 and 35 years old (OR = 0.72, 95%CI [0.55, 0.95]). Among all age categories and education levels, the rates of high intention were much lower than actual acceptance of the maternal pertussis vaccination or COVID-19 vaccination (Fig. 1). Women who refused the maternal pertussis vaccination were highly unlikely to report having a high intention towards receiving the maternal influenza vaccination (OR = 0.39, 95%CI [0.19, 0.79]). Furthermore, being more positive about vaccinations in general was related to a higher intention towards receiving the maternal influenza vaccination (OR = 4.07, 95%CI [3.26, 5.07]).

**Table 2:** Univariate associations with maternal pertussis vaccination behaviour (*accepted/refused)*, COVID-19 vaccination behaviour (*accepted/refused*) and maternal influenza vaccination intention (*low intention/high intention*)

	Maternal pertussis vaccination behaviour					Maternal COVID-19 vaccination behaviour					Maternal influenza vaccination intention				
	Acceptors (*N* = 1294)		Refusers (*N* = 67)			Acceptors (*N* = 787)		Refusers (*N* = 574)			Low Intention (*N* = 981)		High Intention (*N* = 380)		
**Demographic factors**	**n/N**	**%**	**n/N**	**%**	**Unadjusted OR** **(95% CI)**	**n/N**	**%**	**n/N**	**%**	**Unadjusted OR** **(95% CI)**	**n/N**	**%**	**n/N**	**%**	**Unadjusted OR** **(95% CI)**
Age															
*18–24 years* *25–30 years* *31–35 years* *36–40 years* *40 + years*	43/1294 506/1294 553/1294 169/1294 23/1294	3.3 39.1 42.3 13.1 1.8	7/67 26/67 23/67 9/67 2/67	10.4 38.8 34.3 13.4 3.4	0.26 (0.10–0.63)*** 0.81 (0.46–1.44) Ref. 0.78 (0.36–1.72) 0.48 (0.11–2.15)	20/787 271/787 365/787 116/787 15/787	2.5 34.4 46.4 14.7 1.9	30/574 261/574 211/574 62/574 10/574	5.2 45.5 36.8 10.8 1.7	0.39 (0.21–0.70)*** 0.60 (0.47–0.76)*** Ref. 1.08 (0.76–1.54) 0.87 (0.38–1.97)	36/981 411/981 409/981 109/981 17/981	3.7 41.9 41.7 11.0 1.7	14/380 121/380 167/380 70/380 8/380	3.7 31.8 43.9 18.4 2.1	0.95 (0.50–1.81) 0.72 (0.55–0.95)** Ref. 1.59 (1.12–2.25)** 1.15 (0.49–2.72)
Education															
*Low* *Intermediate* *High*	45/1294 369/1294 880/1294	3.5 28.5 68.0	12/67 26/67 29/67	17.9 38.8 43.3	Ref. 3.79 (1.79–8.202*** 8.09 (3.88–16.90)***	26/787 173/787 588/787	3.3 22.0 74.7	31/574 222/574 321/574	5.4 38.7 55.9	Ref. 0.93 (0.53–1.62) 2.18 (1.27–3.74)***	46/981 324/981 611/981	4.7 33.0 62.3	11/380 71/380 298/380	2.9 18.7 78.4	Ref. 0.92 (0.45–1.86) 2.04 (1.04–4.00)**
Country of birth															
*Dutch* *Other*	1230/1294 64/1294	95.1 4.9	56/67 11/67	83.6 16.4	Ref. 0.27 (0.13–0.53)***	748/787 39/787	95.0 5.0	538/574 36/574	93.7 6.3	Ref. 0.78 (0.49–1.24)	934/981 47/981	95.2 4.8	352/380 28/380	92.6 7.4	Ref. 1.58 (0.98–2.56)
**Personal factors**	**n/N**	**%**	**n/N**	**%**	**Unadjusted OR ** **(95% CI)**	**n/N**	**%**	**n/N**	**%**	**Unadjusted OR ** **(95% CI)**	**n/N**	**%**	**n/N**	**%**	**Unadjusted OR ** **(95% CI)**
Pertussis vaccination															
*Yes* *No*	- -	- -	- -	- -	- -	773/787 14/787	98.2 1.8	521/574 53/574	90.8 9.2	Ref. 0.18 (0.10–0.32)***	923/981 58/981	94.1 5.9	371/380 9/380	97.6 2.4	Ref. 0.39 (0.19–0.79)***
**Attitudinal factors**	**Mean**	**SD**	**Mean**	**SD**	**Unadjusted OR ** **(95% CI)**	**Mean**	**SD**	**Mean**	**SD**	**Unadjusted OR ** **(95% CI)**	**Mean**	**SD**	**Mean**	**SD**	**Unadjusted OR ** **(95% CI)**
General beliefs	3.88	0.65	3.10	0.89	4.60 ( 3.20–6.60)***	4.04	0.60	3.56	0.70	3.14 (2.60–3.79)***	3.69	0.66	4.23	0.60	4.07 (3.26–5.07)***

**= *p* < 0.05; ***= *p* < 0.01

### Reasons for accepting or refusing maternal influenza vaccination

Main reasons for expected acceptance of the maternal influenza vaccination were: receiving a recommendation from an obstetrician/gynaecologist (63%), protection of the unborn child (58%), protection of the mother (54%), safety of the vaccination (46%) and following recommendations from the government (43%) (Table 3). Main reasons for expected refusal of the maternal influenza vaccination were: belief in low severity of influenza (40%), vaccination not perceived as necessary (e.g. good health, usually don’t get the vaccination) (22%), too many vaccinations during pregnancy (19%) and low effectiveness of the influenza vaccination (17%).

**Table 3:** Main reasons for predicted maternal influenza acceptance and refusal

Predicted maternal influenza acceptance	Predicted maternal influenza refusal
Receiving a recommendation from HCW (63.2%)	Influenza is not a severe disease (39.7%)
The vaccination is protecting the unborn child (58.3%)	The vaccination is not necessary (good health) (21.6%)
The vaccination is protecting the mother (53.8%)	Too many vaccinations during pregnancy (18.8%)
The vaccination is safe during pregnancy (46.2%)	Low effectiveness of influenza vaccination (17.2%)
Receiving a recommendation from the government (43.3%)	Against all vaccinations during pregnancy (4.4%)
Influenza is a severe disease for the newborn (23.2%)	Not enough information about advantages and disadvantages (3.0%)
Other countries already implemented this vaccination (11.3%)	Fear of or experienced many side effects of the influenza vaccination (2.2%)

## Discussion

This study assessed vaccination behaviour of both the pertussis and COVID-19 vaccination during pregnancy and the intention towards accepting the maternal influenza vaccination within the same study population. In this study, almost all women (95%) were vaccinated against pertussis during pregnancy, while almost two-third were vaccinated against COVID-19 during pregnancy (58%) and almost one-third (28%) had a positive intention towards receiving the maternal influenza vaccination. This indicates that pregnant women are more likely to accept the maternal pertussis vaccination compared to the maternal COVID-19 and influenza vaccination.

### Factors that influence differences in acceptance between the maternal vaccinations

Since the introduction of the maternal pertussis vaccination in the Dutch National Immunisation Program (NIP), several campaigns have been launched to provide information about this vaccination, thereby increasing awareness among citizens. Previous studies have established that greater awareness, of maternal vaccinations in general and their effectiveness, was associated with increased maternal vaccination acceptance [28–30]. In addition, these campaigns can also increase awareness among healthcare professionals (HCPs), which makes them more likely to discuss the maternal pertussis vaccination with pregnant women. Because the maternal influenza vaccination is not yet introduced in the Dutch NIP and there are no campaigns to provide information about this vaccination, HCPs are less likely to discuss the maternal influenza vaccination with pregnant women, let alone recommend it. Nevertheless, previous studies have highlighted the importance of receiving a positive advice from HCPs to increase maternal vaccination acceptance [1, 16–18, 30]. We expect that the campaigns about the maternal pertussis vaccination, and the absence of campaigns about the maternal COVID-19 and influenza vaccination, partly explain the differences in acceptance between the three maternal vaccinations.

Next to lacking recommendations and low awareness, the perceived need to protect the newborn might be a reason for the differences in acceptance between the three maternal vaccinations. Pregnant women who believe that the maternal vaccination will help to protect their newborn, are more likely to get vaccinated during pregnancy. A previous study from Anraad et al. [12] confirmed the importance of outcome expectations by showing a strong correlation between outcome expectations and maternal pertussis vaccination intention. In the case of vaccination behaviour, these outcome expectations are mainly related to the perceived and proven effectiveness of the vaccination [12, 31]. Studies have shown that the maternal pertussis vaccination results in 90% fewer cases of pertussis among newborn, while the maternal influenza vaccination results in 48% fewer cases of influenza among newborn [32–35]. Pregnant women, who do not want to accept all maternal vaccinations, might choose to accept the vaccination with the highest effectiveness. We recommend that future campaigns about the maternal influenza vaccination should focus on increasing awareness about the vaccination, the potential severity of influenza and the fact that the vaccination is both preventive for the pregnant woman and the newborn.

### Factors that influence maternal vaccination acceptance in general

Notwithstanding the differences in acceptance between the three maternal vaccinations, our results show that young maternal age and low educational level were associated with lower maternal vaccination acceptance in general, which is in line with previous studies [15, 16]. Regarding the maternal influenza vaccination, healthy younger women do normally not belong to the risk groups eligible for the regular influenza vaccination. This might cause them to feel less vulnerable, even during pregnancy, making them consider the vaccination is not necessary. This is also the second most frequently cited reason for refusal of the maternal influenza vaccination in our study. However, the association between younger maternal age and vaccination uptake can be affected by the fact that 26.0% of the women aged 18–24 years had a low education level. A previous study from Laenen et al. [36] describes that women with a higher education level might have better access to factual information about the vaccinations, which can explain the association between education level and maternal vaccine acceptance.

Next to younger maternal age and lower education level, maternal pertussis vaccination refusers were also more likely to refuse the COVID-19 vaccination and have a negative intention towards accepting the maternal influenza vaccination. Kilich et al. [30] demonstrated that previous vaccination behaviour had a strong influence on maternal vaccine acceptance, which can possibly explain why maternal pertussis vaccination refusers were more likely to refuse other maternal vaccinations. In addition, for all three vaccinations, refusers, or women with a low intention, scored lower on general vaccination beliefs. This implies that refusers are in general less positive about vaccinations, including maternal vaccinations. These general vaccination beliefs seem to be formed by trust in government, industry and science and nowadays become more important in predicting vaccination acceptance [31]. Previous studies have shown that government trust was associated with compliance with recommendations, such as accepting vaccinations [37, 38]. Luckily, trust can be improved by clear communication about relevant vulnerabilities and possible risks of the disease and the side-effects of vaccination [38]. Future vaccination campaigns should include information about the disease, including vulnerabilities, and about the vaccination, including its positive effects but also its potential side-effects.

### Generalizability

Some selection bias might have played a role in this study despite efforts to reach the most representative sample of Dutch pregnant women and mothers. Especially women of younger age, with a lower education level, of non-Dutch origin and maternal pertussis vaccination refusers seem to have participated less. However, most participants were aged from 25 to 35 years old, comparable with the reference group of pregnant women in the Netherlands [27]. Regarding education level, 14% of women aged 20–25 years had a low education level in 2019 [39] and 11% of women aged 25–45 years had a low education level in 2021 in the Netherlands [40]. In our study, the amount of women with a low education level is lower, namely 4.2%. This is also the case for women of non-Dutch origin. In 2021, 19% of women that gave birth to a child in the Netherlands was born outside the Netherlands [41]. The small amount of women of non-Dutch origin in our sample, can be explained by the use of only Dutch-language questionnaires, making it difficult for women with a lack of proficiency in Dutch to participate. The use of assisted questionnaires at one Youth Health Care location increased the response among these groups. Especially lower educated participants have difficulties in filling in online questionnaires, which makes it less easy to reach them with our general invitation leaflet and online survey. Future studies might focus more on invitation in person, using multiple languages and assisted questionnaire in order to reach a more representative sample.

The acceptance rate of the maternal pertussis vaccination in this study is much higher compared to the general uptake in the Netherlands: 95% versus 66%. This might be explained by a low response rate among refusers. In line with our previous study, a questionnaire study about the MenACWY vaccination for adolescents, vaccine refusers were very hard to reach and they were less likely to fill in the questionnaire [42]. In addition, inviting pregnant women after their pertussis vaccination appointment was part of our recruitment strategy, which might explain the high number of women that were vaccinated against pertussis during pregnancy. Recruitment through assisted questionnaires resulted in a local increase in reaching maternal pertussis refusers, representing 21% of our response by refusers. We are convinced that this particular way of recruiting participants helps to include hard-to-reach groups and has great potential for future studies.

With regards to COVID-19 vaccine acceptance during pregnancy, similar results were reported in a previous study from Canada [43]. Besides, like other studies, the acceptance of COVID-19 vaccination among pregnant women in the Netherlands is lower compared to the general population of reproductive age: 70% of Dutch citizens between the age of 18 and 39 accepted at least two COVID-19 vaccinations [20, 44, 45]. During pregnancy, women might be more cautious about certain behaviours, such as vaccination and taking medication, based on the additional responsibility for their baby and weight of potential side effects of vaccination [18].

While the acceptance rate of COVID-19 vaccination during pregnancy in this study is consistent with previous studies, our study demonstrates that less than one-third (28%) of the women indicated having a positive intention towards maternal influenza, which is lower compared to the uptake in other countries. This might be explained by the fact that the maternal influenza vaccination isn’t part of the Dutch National Immunisation program yet. Vaccinations that are not supported by the national government may be less known, hence less accepted. Besides, implementing a vaccination in the national immunisation program, and launching campaigns, might affect social norm because the vaccination is then seen as ‘standard’. This might influence vaccine acceptance. Therefore, we expect that the actual acceptance of the maternal influenza vaccination might be somewhat higher when the vaccination is a reliant part of the national immunisation program. Nevertheless, we expect that maternal influenza acceptance will not be much higher as the current overall uptake in influenza risk groups in the Netherlands remains below 60%, even after national campaigns [46]. Moreover, among Dutch residents who are eligible for influenza vaccination and are - like pregnant women - below the age of 60 years old, uptake was only 26% in 2020, which is comparable with the percentage of women with positive intention in our study [46].

### Strengths

To the best of our knowledge, this is the first study examining vaccination behaviour or intention of three different maternal vaccinations. Therefore, the study provides insight into specific subgroups that are more likely to refuse maternal vaccinations and shows that existing recommendations and campaigns might facilitate maternal vaccination uptake. This information is useful for improving future maternal vaccination campaigns.

## Conclusion

While increasing maternal vaccination coverage remains a worldwide challenge, this study showed that younger and lower-educated pregnant women were less likely to accept maternal vaccinations. Continuous efforts, such as vaccination campaigns focusing on the severity of diseases that are prevented, are needed to increase maternal vaccine acceptance in these subgroups. Besides, we expect that differences in vaccination coverage between the three maternal vaccinations might be explained by existing recommendations, campaigns and whether the vaccination is part of a national immunisation program.

## Electronic supplementary material

Below is the link to the electronic supplementary material.

**Additional file 1**: Supplementary Table 1.
Overview of questions used to measure constructs.

## Data Availability

The datasets generated and/or analysed during the current study are not publicly available due to the fact that the data of this study contain potentially identifying and sensitive participant information and that publicly sharing the data would not be in accordance with participant’s consent obtained for this study but are available from the corresponding author on reasonable request.

## Abbreviations

ACOG: American College of Obstetricians and Gynecologist
COVID-19: Coronavirus Disease 2019
MenACWY: Meningococcal ACWY
US: United States
WHO: World Health Organization
