# Supplementary material for: Acceptance of vaccination against pertussis, COVID-19 and influenza during pregnancy: a cross-sectional study
Source: BMC Pregnancy Childbirth. 2023 Mar 30;23:219. doi: 10.1186/s12884-023-05505-9 (PMC10061389; doi:10.1186/s12884-023-05505-9)
Supplement: Supplementary file 1 — Additional file 1: Supplementary Table 1. Overview of questions used to measure constructs. [file 12884_2023_5505_MOESM1_ESM.docx]

| **Supplementary table 1: Overview of questions used to measure constructs** | | |
| --- | --- | --- |
| **Construct** | **Question** | **Answer option** |
| Age | What is your age? | - 18 - 24 years - 25 – 30 years - 31 – 35 years - 36 – 40 years - Older than 40 years |
| Education | What is your highest completed education? | - No education (primary school not completed) - Primary education (primary school, special primary education) - Pre-secondary vocational education (vmbo) - Secondary education (havo/vwo) - Intermediate vocational education (mbo1) - Intermediate vocational education (mbo2, mbo3, mbo4) - Higher vocational education (HBO) - University education (WO) - Other, namely…. - Don’t want to say |
| Country of birth | What is your country of birth? | - Netherlands - Western country - Non-Western country |
| General beliefs | 1. Vaccinations are important for staying healthy’  2. In general, going through a disease is better than vaccinating against it  3. There are fewer infectious diseases in the Netherlands because of the National Immunisation Program | 1. Totally disagree 2. Disagree 3. Neutral 4. Agree 5. Totally agree |
| MPV behaviour | Did you accept the maternal pertussis vaccination during pregnancy? | - Yes - No |
| COVID-19 behaviour | Did you accept the /COVID-19 vaccination during pregnancy? | - Yes - No |
| Influenza intention | If the influenza vaccination was offered to pregnant women right now, would you accept it? | - No, never - Probably not - Maybe yes, maybe no - Probably yes - Yes, always |
| Reasons refusal influenza | What are reasons for you to refuse the influenza vaccination during pregnancy? | Multiple choice   - I prefer not to have two vaccinations during pregnancy - Influenza is not a serious disease - I am against vaccinations during pregnancy - I think the vaccination is not effective - Because of my religion, I am against vaccination - Other, namely…. |
| Reasons acceptance influenza | What are reasons for you to accept the influenza vaccination during pregnancy? | Multiple choice   - I follow the government's advice on vaccinations - I follow the advice of my midwife/gynaecologist regarding vaccinations - The vaccination has benefits for the unborn child - The vaccination has benefits for the pregnant woman herself - I think influenza is dangerous for newborn - Other countries already offer influenza vaccination during pregnancy - The vaccination is safe during pregnancy - Other, namely… |
